# Supplementary material for: Machine prescription for chronic migraine
Source: Brain Commun. 2022 Mar 10;4(3):fcac059. doi: 10.1093/braincomms/fcac059 (PMC9070525; doi:10.1093/braincomms/fcac059)

Supplementary material

The supplementary material consists of the following:

- Analytic environment description
- Supplementary Table 1 – Detailed description of features and outcomes
- Supplementary Table 2 – Causal multitask gaussian process kernel optimization
- Supplementary Table 3 – Expert panel ranking of treatments
- Supplementary Table 4 – Treatment price estimates
- Supplementary Table 5 – Time-to-response treatment given different strategies to decide the order of treatment trials.
- Supplementary Figure 1 – Flow-chart of study population
- Supplementary Figure 2 – Average treatment effects in training and out-of-sample

Analytic environment

All data management, analysis and figures were made using python 3.7 (python software foundation) using open-source packages. The following dependencies were used docx 0.8.10; GPy 1.9.9; matplotlib 3.1.1; numpy 1.17.2; pandas 0.25.1; predictive-imputer 0.2.0; regex 2019.8.19; scikit-learn 0.22.1; scipy 1.3.1; skopt 0.6; seaborn 0.9.0; umap 0.3.10; and xgboost 0.90.

Supplementary Table 1. Description of modelled demographics, clinical features and treatments.

No	Feature	Feature description
1	Gender	Female or male
2	Age	Age at first contact with the tertiary headache centre and at time of phenotypic description.
3	Headache frequency	Number of days with headache per month (i.e., out of 30 days)
4	Exacerbation intensity	Intensity of headache exacerbations rated on a 0-10 NRS scale.
5	Unilateral headache	Some proportion or duration of headache episodes with unilateral pain.
6	Bilateral headache	Some proportion or duration of headache episodes with bilateral pain.
7	Throbbing quality	Including synonyms of throbbing pain character.
8	Nausea	Nausea associated with the headache.
9	Vomiting	Vomiting associated with the headache.
10	Photophobia	Photophobia associated with the headache.
11	Phonophobia	Phonophobia associated with the headache.
12	Osmophobia	Osmophobia associated with the headache.
13	Motion sensitivity	Motion sensitivity and pain aggravation upon routine physical activity during headache.
14	Difficulty concentrating	Difficulties with concentrating during headache.
15	Vertigo	Vertigo associated with the headache.
16-24	Cranial autonomic symptoms	Nine cranial autonomic symptoms: Conjunctival injection, lacrimation, eyelid oedema, ptosis, nasal block, rhinorrhea, aural fullness, facial sweating, facial redness. Cranial autonomic symptoms were not required to be exclusively unilateral.
25	Restlessness	Restlessness or agitation during headache.
26	Visual aura	Visual symptoms were classified as visual aura in presence of characteristic phenomena such as flashing lights, zig-zag lines, colour changes and scotoma. Blurring of vision or difficulty focusing alone were not termed as visual aura.
27	Sensory aura	Presence of somatosensory phenomena such as numbness, tingling and paraesthesia.
28	Speech aura	Presence of speech symptoms such as word finding difficulties, paraphasia and dysphasia.
29	Motor aura	Presence of motor symptoms such as weakness, facial drop and hemiplegia. Clumsiness alone was not categorized as motor aura.
30	Brainstem aura	Definite brainstem aura as categorized by ICHD-3.
31	Trigeminal V1 pain site	Pain situated to innervation area of first trigeminal branch.
32	Trigeminal V2 pain site	Pain situated to innervation area of second trigeminal branch.
33	Trigeminal V3 pain site	Pain situated to innervation area of third trigeminal branch.
34	Occipital pain site	Pain situated occipitally.
35	Neck and shoulder pain site	Pain primarily situated at the neck and/or shoulder.
36	Trigeminal V1 pain radiation	Pain radiating to innervation area of first trigeminal branch.
37	Trigeminal V2 pain radiation	Pain radiating to innervation area of second trigeminal branch.
38	Trigeminal V3 pain radiation	Pain radiating to innervation area of third trigeminal branch.
39	Occipital pain	Pain radiating to the occiput.
40	Neck and shoulder pain	Pain radiating to the neck and/or shoulder.
41	Stress trigger	Headaches triggered by subjective feeling of stress or mental exertion
42	Menstruation trigger	Headaches triggered by menstruation or ovulation.
43	Sleep related triggers	Headaches triggered by sleep deprivation or sleeping in.
44	Food and drink triggers	Headaches triggered by dietary factors such as caffeine, alcohol, specific foods or feeling hungry or dehydrated.
45	Alcohol trigger	Headaches triggered by alcohol
46	Sensory triggers	Headaches triggered by specific sensory stimuli such as lights, noises and smells.
47	Weather and environmental triggers	Headaches triggered by weather changes, warm or cold weather.
48	Exertional triggers	Headaches triggered by physical overexertion.
49	Family history of migraine	Family history of migraine.
50	Family history of hypermobility	Family history of hypermobility (including Ehlers Danlos syndrome).
51-76	Comorbidities	Medication overuse headache, hypermobility (including Ehlers Danlos Syndrome), Cluster headache, SUNCT/SUNA, Cervicogenic headache, Tension type headache, any type of head or facial neuralgia, Hemicrania continua, Paroxysmal hemicrania, Orthostatic headache, Intracranial idiopathic hypertension, Epilepsy, Postural orthostatic tachycardia syndrome, Anxiety, Depression, Back pain, Chronic fatigue syndrome, Temporomandibular joint dysfunction, Irritable bowel syndrome, (Arnold) Chiari malformation, Gastrointestinal dysfunction, Posttraumatic migraine, Vestibular migraine, Hemiplegic migraine, Head injury, CVD risk factors (ischemic heart disease, hypertension, diabetes, overweight).

No	Treatment group	Description
1	Tricyclic antidepressants	Amitriptyline, Nortriptyline, Dosulepin, Imipramine
2	Serotonin Noradrenaline Reuptake Inhibitors	Duloxetine, Mirtazapine, Venlafaxine
3	Betablockers	Propranolol, Atenolol, Bisoprolol, Metoprolol
4	Angiotensin receptor blockers	Candesartan
5	Calcium channel blockers	Flunarizine
6	Topiramate	Topiramate
7	Valproate	Valproate
8	Serotonergic agents	Methysergide, Pizotifen
9	Acupuncture	Acupuncture
10	Botox	Botox according to the PREEMPT regime

Supplementary Table 2. Causal Multitask Gaussian Process kernel optimization. The optimal—and final reported—model is highlighted in bold. The discounted cumulative gain (DCG) is a scoring metric for comparing a predicted ranking of a set of items to a "ground truth" ranking defined by the comparative relevance of each item. In our case, the item is a treatment, and its relevance is the propensity to produce a positive effect. Thus, the more accurate the ordering of treatments in terms of relevance (positive effect), the higher the DCG. Missing values for the "ground truth" were replaced by the mean. The normalized discounted cumulative gain (NDCG) is a normalization of DCG that adjusts for different lengths of ranked lists. Despite slightly poorer performance on the validation set, the counterfactual Matern kernel was chosen, as this provided validation treatment effect estimates more faithful to the training estimates.

Modality	Kernel	DCG	NDCG
Counterfactual	RBF	1.537	0.514
**Counterfactual**	**Matern**	**1.534**	**0.511**
Multitask	RBF	1.534	0.512
Multitask	Matern	1.527	0.508
VirtualTwin	RBF	1.520	0.505
VirtualTwin	Matern	1.517	0.503

Supplementary Table 3. Expert panel ranking of treatments. Twenty-five UK headache specialists were asked to ascertain the order in which they would use the following drugs, assuming the patients has no contraindications of preferences: betablockers, candesartan, flunarizine, pizotifen, serotonin, noradrenaline reuptake inhibitors, topiramate, tricyclic antidepressants and valproate. 23 of 25 specialist replied to the query. The consensus ordering of the treatments was based on the mean rank.

	PS	GL	SM	CD	AT	JG	KM	N	MM	JA	BD	SK	AT	FA	GK	SW	JV	RF	AC	NN	MK	SG	BA	**Mean**	**Median**	**Rank**
DRUG RANKING																										
Betablocker	2	2	1	1	2	1	4	2	1	5	2	3	1	1	1	3	2	6	2	1	2	2	2	**2,13**	**2**	**1**
Candesartan	6	5	5	3	5	2	3	4	2	1	7	1	3	3	4	1	7	7	4	4	1	4	1	**3,61**	**4**	**4**
Flunarizine	5	4	6	5	6	5	7	6	6	6	3	8	5	6	8	5	8	3	7	6	6	8	6	**5,87**	**6**	**5**
Pizotifen	4	6	3	7	7	6	6	5	8	7	6	6	6	7	7	6	5	8	6	7	5	6	8	**6,17**	**6**	**6**
SNRIs	8	8	8	8	8	8	5	8	4	4	8	5	8	5	5	7	4	4	5	8	7	5	5	**6,30**	**7**	**7**
Topiramate	1	3	4	2	3	4	2	3	5	3	1	4	4	4	3	4	1	2	3	2	4	3	4	**3,00**	**3**	**3**
Tricyclic antidepressants	3	1	2	4	1	3	1	1	3	2	4	2	2	2	2	2	3	1	1	3	3	1	3	**2,17**	**2**	**2**
Valproate	7	7	7	6	4	7	8	7	7	8	5	7	7	8	6	8	6	5	8	5	8	7	7	**6,74**	**7**	**8**

Supplementary Table 4. Treatment cost estimates. Based on BNF drug tariff prices (June 2020) except where noted. Costs are given for one-month treatment in GBP. Acupuncture and botox prices represent three months treatment.

Treatment	Description	Cost
**TRICYCLIC ANTIDEPRESSANTS (75 mg daily)**		
**Amitriptyline**		
Low estimate	Amitriptyline hydrochloride 50+25mg tablets	£2.71
High estimate	Amitriptyline hydrochloride 50+25mg tablets	£2.71
**Nortriptyline**		
Low estimate	Nortriptyline hydrochloride 50+25mg tablets	£42.23
High estimate	Nortriptyline hydrochloride 50+25mg tablets	£42.23
**Dosulepin**		
Low estimate	Dosulepin hydrochloride 75mg tablets	£17.17
High estimate	Dosulepin hydrochloride 75mg tablets	£17.17
**Imipramine**		
Low estimate	Imipramine hydrochloride 3*25mg tablets	£3.69
High estimate	Imipramine hydrochloride 3*25mg tablets	£3.69
**SEROTONIN NORADRENALINE REUPTAKE INHIBITORS**		
**Duloxetine (90 mg)**		
Low estimate	Duloxetine hydrochloride 30+60mg capsules	£4.17
High estimate	Duloxetine hydrochloride 30+60mg capsules	£4.17
**Mirtazapine (45 mg)**		
Low estimate	Mirtazapine 45mg tablets	£1.94
High estimate	Mirtazapine 45mg orodispersable tablet	£2.18
**Venlafaxine (300 mg)**		
Low estimate	Venlafaxine hydrochloride 2*150mg capsules	£7.8
High estimate	Venlafaxine hydrochloride 2*150mg capsules	£7.8
**BETABLOCKERS**		
**Propranolol (160 mg)**		
Low estimate	Propranolol 160mg capsules	£4.88
High estimate	Propranolol 160mg tablets	£5.88
**Atenolol (100 mg)**		
Low estimate	Atenolol 100mg tablets	£0.82
High estimate	Atenolol 100mg tablets	£0.82
**Bisoprolol (10 mg)**		
Low estimate	Bisoprolol fumarate 10mg tablets	£0.98
High estimate	Bisoprolol fumarate 10mg tablets	£0.98
**Metoprolol (100 mg)**		
Low estimate	Metoprolol tartrate 100mg tablets	£3.87
High estimate	Metoprolol tartrate 100mg tablets	£3.87
**ANGIOTENSIN RECEPTOR BLOCKERS**		
**Candesartan (32 mg)**		
Low estimate	Candesartan cilextil 32mg tablets	£2.09
High estimate	Candesartan cilextil 32mg tablets	£2.09
**CALCIUM CHANNEL BLOCKERS**		
**Flunarizine (10 mg)**		
Low estimate	Flunarizine hydrochloride 5m capsule	£68.92[a]
High estimate	Flunarizine hydrochloride 5m capsule	£68.92[a]
**TOPIRAMATE**		
**Topiramate (100 mg)**		
Low estimate	Topiramate 100mg tablets	£19.72
High estimate	Topiramate 100mg tablets	£19.72
**VALPROATE**		
**Valproate (1.5 g)**		
Low estimate	Sodium valproate 500+1000mg modified-release granules	£51.00
High estimate	Sodium valproate 500+1000mg modified release tablets	£62.00
**SEROTONERGIC AGENTS**		
**Pizotifen (4.5 mg)**		
Low estimate	Pizotifen hydrogen malate 3*1.5mg tablet	£5.52
High estimate	Pizotifen hydrogen malate 3*1.5mg tablet	£5.52
**Methysergide (not available)**		
Low estimate[b]		
High estimate[b]		
**ACUPUNCTURE**		
**Acupuncture**		
Low estimate	10 sessions over a 3-month period	£275[c]
High estimate	10 sessions over a 3-month period	£520[c]
**BOTOX**		
**Botox**		
Low estimate	Botulinum toxin type A 125 unit 349.40	£349.40[d]
High estimate	Botulinum toxin type A 200 unit 349.40	£349.40[d]

[a] Not available from BNF drug tariffs. Captured from NICE guidelines on migraine prophylaxis where the NHS prescription cost analysis for England 2013 estimates a cost of £68.92 per unit.

[b] Not available from BNF drug tariffs.

[c] Not available from BNF drug tariffs. Pricing captured from the British Acupuncture Council. Estimates based on 10 sessions over a 3-month period. Low price estimate calculated as £50+(£25*9) and high estimate as £70+(£50*9)

[d] 3-months treatment expenses. https://www.nice.org.uk/guidance/ta260/chapter/2-The-technology

Supplementary Table 5. Time-to-response given different strategies to decide the order of treatment trials. For the restricted machine learning ordering, expert panel ordering, and all guideline orderings, onabotulinumtoxinA was set as the fourth treatment choice as per UK National Health Service regulations. Guideline recommendation 1 was constructed by picking three random of the evidence-based oral preventives suggested in at least one guideline (tricyclic antidepressants, serotonin noradrenaline reuptake inhibitors, betablockers, candesartan, topiramate, valproate and flunarizine) followed by onabotulinumtoxinA. Guideline recommendation 2 was based on picking two random among betablocker, candesartan, tricyclic antidepressants and serotonin noradrenaline reuptake inhibitors, followed by one random of topiramate, valproate and flunarizine, followed by onabotulinumtoxinA. The National Institute for Health and Care Excellence (NICE) guideline sequence was based on the recommendation of trying each of betablockers, topiramate and tricyclic antidepressants (order not specified), followed by onabotulinumtoxinA, followed by random ordering. The final expert panel ordering was based on an aggregate of 23 UK headache specialists asked to order the treatments based on a general understanding of efficacy and adverse events. Legend: SD=standard deviation; ML=machine learning; MD=mean difference; CI=confidence interval; NICE=National Institute for Health and Care Excellence

Strategy to decide the order of treatment trials	Mean (SD) months to completion of first effective treatment (n=253)	vs ML model (MD, 95% CI, p-value)	vs restricted ML model (MD, 95% CI, p-value)
Machine prescription	7.12 (1.59)	..	..
Machine prescription restricting onabotulinumtoxinA to fourth treatment choice	6.85 (1.48)	..	..
Guideline recommended ordering 1	10.60 (1.30)	-3.477 (-3.730 to -3.224), p<0.0001	-3.750 (-3.993 to -3.507), p<0.0001
Guideline recommended ordering 2	10.46 (1.51)	-3.343 (-3.612 to -3.071), p<0.0001	-3.615 (-3.876 to -3.353), p<0.0001
NICE guideline ordering	10.48 (1.64)	-3.364 (-3.646 to -3.082), p<0.0001	-3.637 (-3.910 to -3.364), p<0.0001
Expert panel ordering	10.87 (1.99)	-3.748 (-4.063 to -3.434), p<0.0001	-4.021 (-4.382 to -3.715), p<0.0001
Random ordering	10.86 (1.11)	-3.737 (-3.976 to -3.498), p<0.0001	-4.010 (-4.238 to -3.782), p<0.0001
Ordering by price (low drug tariff estimate)	10.92 (2.58)	-3.803 (-4.178 to -3.429), p<0.0001	-3.791 (-4.734 to -2.874), p<0.0001
Ordering by price (high drug tariff estimate)	10.84 (2.48)	-3.718 (-4.082 to -3.354), p<0.0001	-3.991 (-4.348 to -3.633), p<0.0001

Supplementary Figure 1. Flow-chart of study population. The left side of the flow-chart shows how data was acquired, whereas the right side show what data was unavailable while training the data extraction and machine learning algorithms. The latter is only used as out-of-sample test data. The lower part of the flow-chart with the colored boxes show how the main dataset is divided in train/validation/test splits. The train and validation splits are used for training, optimizing and selecting machine learning models (red and yellow box). This model is finally applied on the unseen test set (green box).

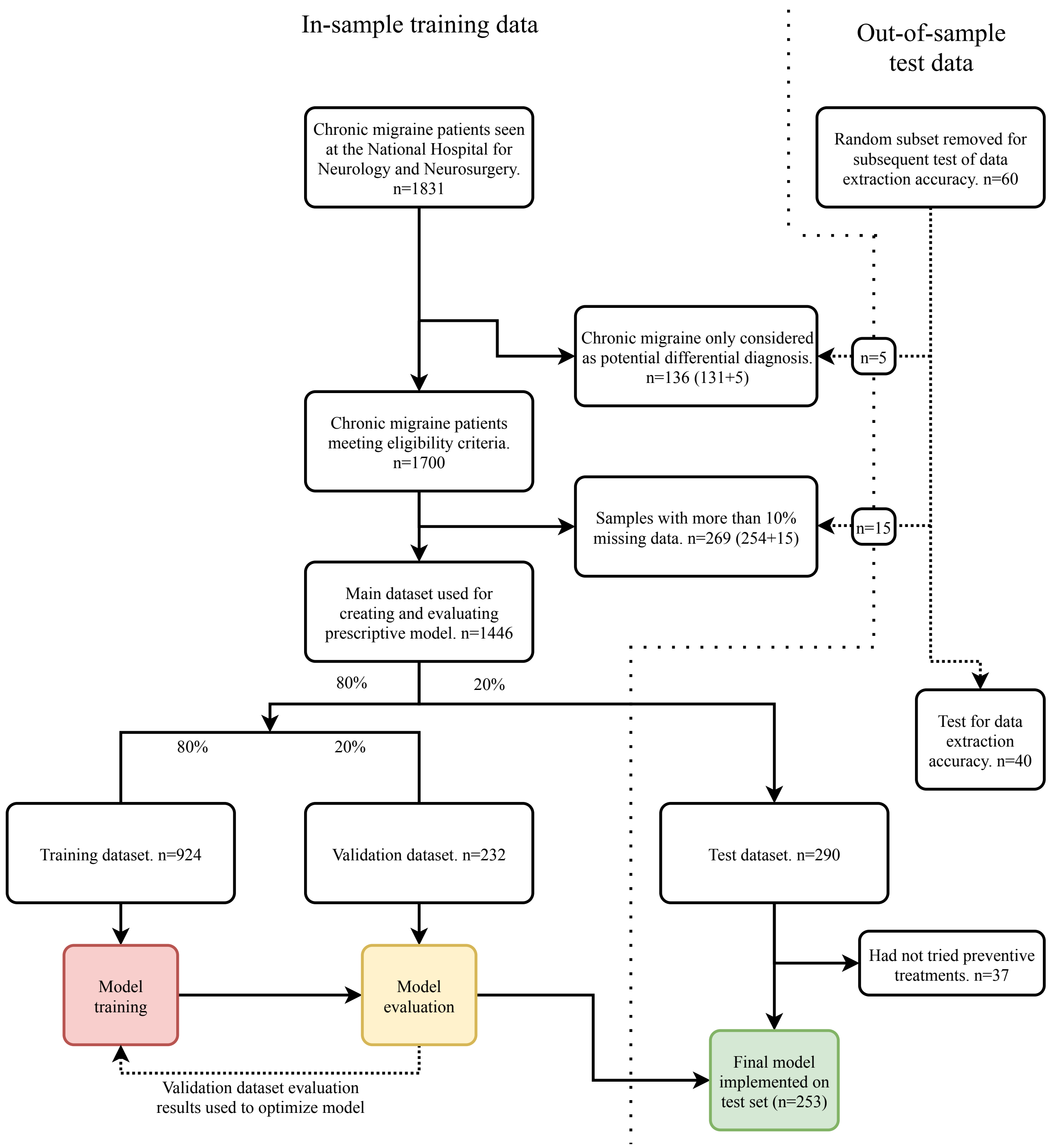

Supplementary Figure 2. Violinplot showing the distribution of the mean of individualized treatment effects across all pairwise comparisons for the in-sample train data (blue, n=924) and the out-of-sample test data (orange, n=253) based on the causal multitask gaussian process model. The blue training data can be interpreted as retrospective treatments effects for a cohort study, while the orange test data can be interpreted as the predicted population treatment effect. Note that the out-of-sample data remain faithful to the training data.

SNRI=serotonin reuptake inhibitors; TCA=tricyclic antidepressants

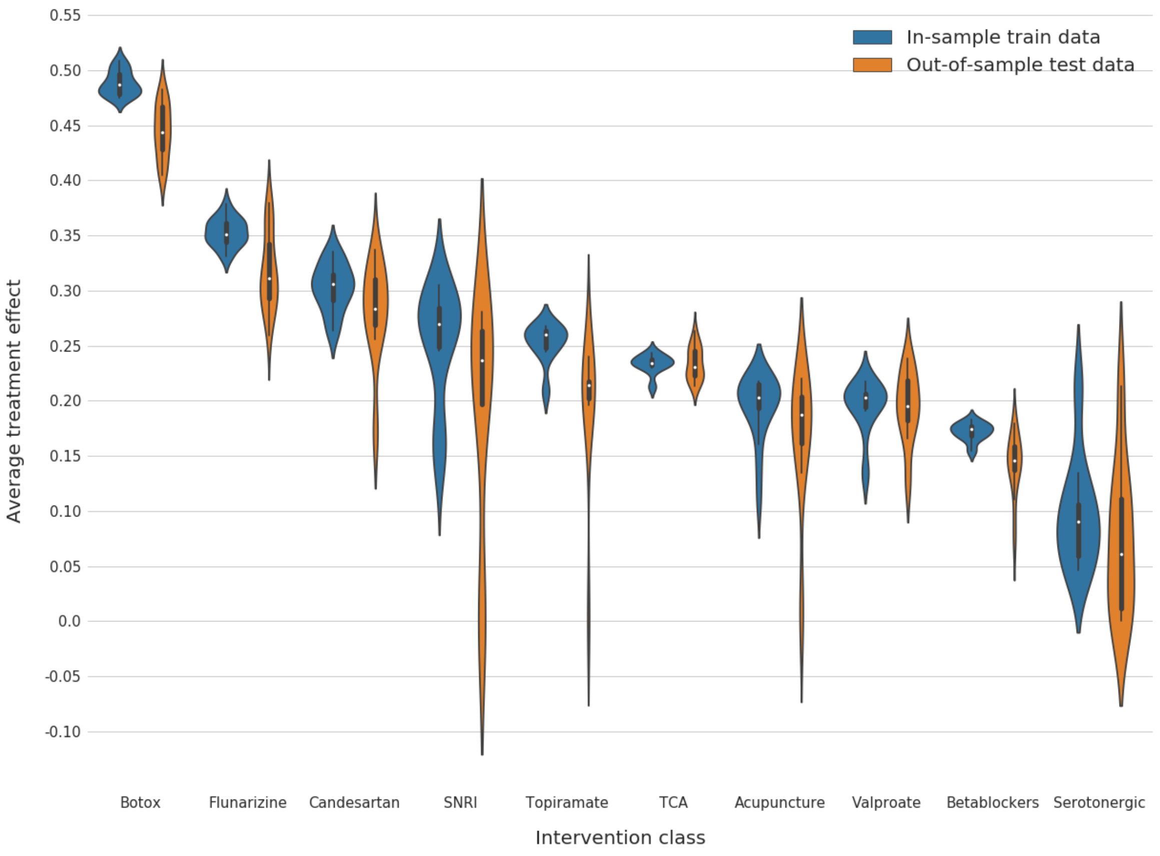

Supplement: fcac059_Supplementary_Data [file fcac059_supplementary_data.zip › Supplementary_material.pdf]
