# Supplementary material for: Machine prescription for chronic migraine
Source: Brain Commun. 2022 Mar 10;4(3):fcac059. doi: 10.1093/braincomms/fcac059 (PMC9070525; doi:10.1093/braincomms/fcac059)
Supplement: fcac059_Supplementary_Data [file Original Submission - Decision Letter.pdf › Original Submission - Decision Letter.pdf]

Original Submission – Decision Letter

22-Dec-2021

Dear Dr. Stubberud,

Thank you for your recent submission BRAINCOM-2021-278
entitled "Machine prescription for chronic migraine" to Brain Communications.

Your manuscript has been reviewed, and while the reviewers had largely positive comments, they have some constructive critiques that I think you could address in a minor revision of the manuscript. Therefore, I invite you to respond to the reviewer(s)' comments and revise your manuscript. The reviewer(s)' comments are included at the bottom of this message.

In addition to the reviewers' comments, please also address our Scientific Editor's comments added below to comply with our requirements for presentation of scientific data at Brain Communications.

Please format your revised manuscript according to our Instructions to Authors https://academic.oup.com/braincomms/pages/General_Instructions – a summary of key points is attached to help with this.

To revise your manuscript, click the link below or log into https://mc.manuscriptcentral.com/braincom and enter your Author Centre, where you will find your manuscript title listed under "Manuscripts with Decisions." Under "Actions," click on "Create a Revision." Your manuscript number will be appended to denote a revision.

*** PLEASE NOTE: This is a two-step process. After clicking on the link, you will be directed to a webpage to confirm. ***

https://mc.manuscriptcentral.com/braincom?URL_MASK=277373194e7b4d868cd11c63890d48ee

Please upload a revised manuscript with the changes you have made shown either in coloured text or using "track changes".

When submitting your revised manuscript, you will be asked to respond to the reviewer(s) comments. Please be as specific as possible in your response to all of the comments.

The editorial team at Brain Communications also aims to increase transparency in scientific publishing and to demonstrate the value that peer review brings to the scientific process. Please therefore consider whether you are willing to have reviewer comments on your paper and your response published as supplementary data with the paper, if it is accepted. This is entirely optional and will only be done if both the authors and reviewers agree. When submitting your revision you will have the opportunity to update the answer you entered previously.

IMPORTANT: Your original files are available to you when you upload your revised manuscript. Please delete any redundant files before completing the submission.

Because we are trying to facilitate timely publication of manuscripts submitted to Brain Communications, we would appreciate your revised manuscript as soon as possible. If it is not possible for you to resubmit within 3 months, the option to submit a revision will expire, and we would then consider your revised paper as a new submission. However, if you need a little more time, please get in touch and we can extend this deadline for you.

Once again, thank you for submitting your manuscript to Brain Communications and I look forward to receiving your revision.

Best wishes

Dr. Graciela Muniz Terrera
Associate Editor, Brain Communications

Professor Tara Spires-Jones
Founding Editor, Brain Communications

Reviewer(s)' Comments to Author:

Reviewer: 1

Comments to the Author
Nice piece of work on generalizable migraine treatment prediction, especially given the complexity of such a prediction for different varieties/severities of migraine attacks and yet unknown underlying mechanism in the brain. The authors need to clarify a few points and address a couple of minor issues before this paper can be considered for publication:

1- Page 5, line 54: please write the full form of "(MM)" and/or define it somewhere (e.g., footnote)
2- Page 5, lines 58-60: "....sufficiently complete structured clinical phenotypic...": please refrain from using qualitative adjectives such as "sufficiently" here. Please quantify this criterion of including the patients in the study.
3- Page 6, line 30: Please clearly define the "headache days" and specify the number of observation days: headache days over at least three months? Up to how many days? The length of this period affects the effectiveness of the agents.
4- Page 6, line 44: what do you mean by "missingness" this here? Please clarify.
5- Page 6, line 56: what does manual extraction mean? not clear, please clarify.
6- Page 7, line 11: what do you mean by 10% missing data? Does it mean the number of missing features?
7- Page 8, line 49: why logistic regression was used? please provide some justification and comparison with other regression models, if possible.

In all of the figures, the font size is too small (axis labels, titles, etc.). Please make it larger.

Best,

Reviewer: 2 (Arun Swaminathan)

Comments to the Author
Very well written paper with innovative and timely use of AI to support diagnostic paradigms. I expect that using such approaches will improve diagnostic accuracy and therapeutic benefit as well.

Comments from the Scientific Editor:

-For analyses reported in figures, please, include in the legends provide details of the statistical analysis performed alongside the quantitative parameters, and the number of data-points for each group analysed in the plots, and if the number is <10 show the single data-points. Please note our policy on pseudoreplication, we do not accept analyses where individual cells are the experimental unit when there is likely biological variability is between animals, culture replicates, etc. Please see instructions for authors for more details. Fort his type of data, we require either that a mean/median be taken per animal/culture replicate followed by statistical analyses or that more sophisticated analyses are performed such as mixed effects modelling which can account for random variables.

-Please supply a graphical abstract. A graphical abstract is a visual representation of the central finding or methodology of the paper. It will be displayed in the table of contents. Authors should strive to make the graphical abstract informative, interesting, visually appealing, and straightforward (no caption needed, no abbreviations except gene names). Please upload as a separate high resolution image file (tiff preferred). The technical requirements for the graphical abstracts are:
  --size of the submitted image: 1200 pixels square at 300dpi
  --font of the text: Arial 12-16 point (smaller fonts will not be legible online)

-Please add an Abbreviated Summary (up to 50 words) of your paper that captures the main purpose and conclusions of the work. The summary should state the main results written in the third person (‘[1st author surname] et al. report that …’) and conclusion. This summary will be used in the contents list online and should not contain any abbreviations other than those on the accepted abbreviations list and accepted gene/protein names, making it accessible to the non-specialist. Please upload it as a Word document. Allowed abbreviations: AIDS; ANOVA; AMPA; ATP; A,T,C,G; CNS; CSF; CT; DNA; ECG; EEG; EMG; GABA; HIV; MRI; NMDA; PET; PCR; RNA.
