# Supplementary material for: Machine prescription for chronic migraine
Source: Brain Commun. 2022 Mar 10;4(3):fcac059. doi: 10.1093/braincomms/fcac059 (PMC9070525; doi:10.1093/braincomms/fcac059)
Supplement: fcac059_Supplementary_Data [file Original Submission-Author's Response(1).pdf › Original Submission-Author's Response(1).pdf]

Response to reviewers and editorial comments

Thank you for considering our manuscript and thanks to both reviewers and the editors for favourable comments. Below are the comments addressed point-by-point.

Reviewer(s)' Comments to Author:

Reviewer: 1

Comments to the Author
Nice piece of work on generalizable migraine treatment prediction, especially given the complexity of such a prediction for different varieties/severities of migraine attacks and yet unknown underlying mechanism in the brain. The authors need to clarify a few points and address a couple of minor issues before this paper can be considered for publication:

1- Page 5, line 54: please write the full form of "(MM)" and/or define it somewhere (e.g., footnote)
Thank you. We have now clarified what is meant here.

2- Page 5, lines 58-60: "....sufficiently complete structured clinical phenotypic...": please refrain from using qualitative adjectives such as "sufficiently" here. Please quantify this criterion of including the patients in the study.
Thank you for the comment. We have now stated that a record using the standard template at the clinic was required for inclusion.

3- Page 6, line 30: Please clearly define the "headache days" and specify the number of observation days: headache days over at least three months? Up to how many days? The length of this period affects the effectiveness of the agents.
On page 6 we have now included a more detailed definition of headache days and the evaluation period.

4- Page 6, line 44: what do you mean by "missingness" this here? Please clarify.
Thank you for pointing this out. We have addressed comment 4 and 6 by stating that subjects missing data for more than 10% of the features were removed from the dataset on page 6 and 7.

5- Page 6, line 56: what does manual extraction mean? not clear, please clarify.
We have now clarified what we mean by manual extraction on page 7.

6- Page 7, line 11: what do you mean by 10% missing data? Does it mean the number of missing features?
Thank you for pointing this out. We have addressed comment 4 and 6 by stating that subjects missing data for more than 10% of the features were removed from the dataset on page 6 and 7.

7- Page 8, line 49: why logistic regression was used? please provide some justification and comparison with other regression models, if possible.

Thank you for commenting on this. Logistic regression was used because there was only one independent variable, and the dependent variable was binary. We found this analysis to be the most suited. Moreover, the logistic regression primarily serves as a supplementary analysis to the discounted cumulative gain for evaluating prediction performance.

In all of the figures, the font size is too small (axis labels, titles, etc.). Please make it larger.
Thank you. We have enlarged all text in all figures. Please let us know if further enlargement is desired.

Best,

Reviewer: 2

Comments to the Author
Very well written paper with innovative and timely use of AI to support diagnostic paradigms. I expect that using such approaches will improve diagnostic accuracy and therapeutic benefit as well.

Comments from the Scientific Editor:

-For analyses reported in figures, please, include in the legends provide details of the statistical analysis performed alongside the quantitative parameters, and the number of data-points for each group analysed in the plots, and if the number is <10 show the single data-points. Please note our policy on pseudoreplication, we do not accept analyses where individual cells are the experimental unit when there is likely biological variability is between animals, culture replicates, etc. Please see instructions for authors for more details. Fort his type of data, we require either that a mean/median be taken per animal/culture replicate followed by statistical analyses or that more sophisticated analyses are performed such as mixed effects modelling which can account for random variables.
We have now included details of the statistical analysis in the figure legends. We believe the present presentation of violin plots does not violate the policy on pseudoreplication.

-Please supply a graphical abstract. A graphical abstract is a visual representation of the central finding or methodology of the paper. It will be displayed in the table of contents. Authors should strive to make the graphical abstract informative, interesting, visually appealing, and straightforward (no caption needed, no abbreviations except gene names). Please upload as a separate high resolution image file (tiff preferred). The technical requirements for the graphical abstracts are:
--size of the submitted image: 1200 pixels square at 300dpi
--font of the text: Arial 12-16 point (smaller fonts will not be legible online)
A graphical abstract is now included in the manuscript and uploaded as a high-resolution image file separately.

-Please add an Abbreviated Summary (up to 50 words) of your paper that captures the main purpose and conclusions of the work. The summary should state the main results written in the third person ('[1st author surname] et al. report that …') and conclusion. This summary

will be used in the contents list online and should not contain any abbreviations other than those on the accepted abbreviations list and accepted gene/protein names, making it accessible to the non-specialist. Please upload it as a Word document. Allowed abbreviations: AIDS; ANOVA; AMPA; ATP; A,T,C,G; CNS; CSF; CT; DNA; ECG; EEG; EMG; GABA; HIV; MRI; NMDA; PET; PCR; RNA.

We have included an abbreviated summary after the abstract.
