# Supplementary material for: Machine prescription for chronic migraine
Source: Brain Commun. 2022 Mar 10;4(3):fcac059. doi: 10.1093/braincomms/fcac059 (PMC9070525; doi:10.1093/braincomms/fcac059)
Supplement: fcac059_Supplementary_Data [file Revision 1 - Decision Letter.pdf › Revision 1 - Decision Letter.pdf]

Revision 1 - Decision Letter

09-Feb-2022

Dear Dr. Stubberud,

It is a pleasure to provisionally accept your manuscript entitled "Machine prescription for chronic migraine" for publication in Brain Communications. Any comments made by the reviewer(s) are included below. We will be in touch soon with a few changes needed to comply with our policies on the presentation of data.

Video abstracts - Brain Communications encourages authors to provide a video abstract to accompany their article. These are hosted on the Brain/Brain Communications YouTube channel. If you are interested in producing a 5-min video abstract to promote your article, then please contact the Editorial Office (brcoms.editorialoffice@oup.com) for further details and guidelines.

Thank you very much for your contribution. On behalf of the Editors of Brain Communications, we look forward to your future contributions to the Journal.

Best wishes

Dr. Graciela Muniz Terrera
Associate Editor, Brain Communications

Professor Tara Spires-Jones
Founding Editor, Brain Communications

Reviewer(s)' Comments to Author:

Reviewer: 2 (Arun Swaminathan)

Comments to the Author
Very well designed study and certainly shows proof of concept for the use of AI in decision making pathways, especially for migraines.

Reviewer: 1

Comments to the Author
All of the reviewers' comments have been carefully addressed in this revised version.
